# Sustainable Materials Containing Biochar Particles: A Review

**DOI:** 10.3390/polym15020343

**Published:** 2023-01-09

**Authors:** Giulia Infurna, Gabriele Caruso, Nadka Tz. Dintcheva

**Affiliations:** Dipartimento di Ingegneria, Università degli Studi di Palermo, Edificio 6, 90128 Palermo, Italy

**Keywords:** biochar particles, sustainable materials, polymers, biopolymers, asphalts

## Abstract

The conversion of polymer waste, food waste, and biomasses through thermochemical decomposition to fuels, syngas, and solid phase, named char/biochar particles, gives a second life to these waste materials, and this process has been widely investigated in the last two decades. The main thermochemical decomposition processes that have been explored are slow, fast, and flash pyrolysis, torrefaction, gasification, and hydrothermal liquefaction, which produce char/biochar particles that differ in their chemical and physical properties, i.e., their carbon-content, CHNOS compositions, porosity, and adsorption ability. Currently, the main proposed applications of the char/biochar particles are in the agricultural sector as fertilizers for soil retirement and water treatment, as well as use as high adsorption particles. Therefore, according to recently published papers, char/biochar particles could be successfully considered for the formulation of sustainable polymer and biopolymer-based composites. Additionally, in the last decade, these particles have also been proposed as suitable fillers for asphalts. Based on these findings, the current review gives a critical overview that highlights the advantages in using these novel particles as suitable additives and fillers, and at the same time, it shows some drawbacks in their use. Adding char/biochar particles in polymers and biopolymers significantly increases their elastic modulus, tensile strength, and flame and oxygen resistance, although composite ductility is significantly penalized. Unfortunately, due to the dark color of the char/biochar particles, all composites show brown-black coloration, and this issue limits the applications.

## 1. Introduction

Nowadays, the conversion of polymer waste, food waste, and biomass aimed at reducing their impact on the environment gives them a second life, and changing from a linear economy to a circular economy is being widely investigated [1,2]. Different thermochemical decomposition processes leading to the recovery of fuels and residual solid phase have been taken into consideration, including those methods that could be profitable for some applications, such as for soil remediation, as additives, for use as synthetic carbonaceous particles, for the formulation of polymer and biopolymer-based composites, as additives for asphalts, etc.

**Figure 1:**
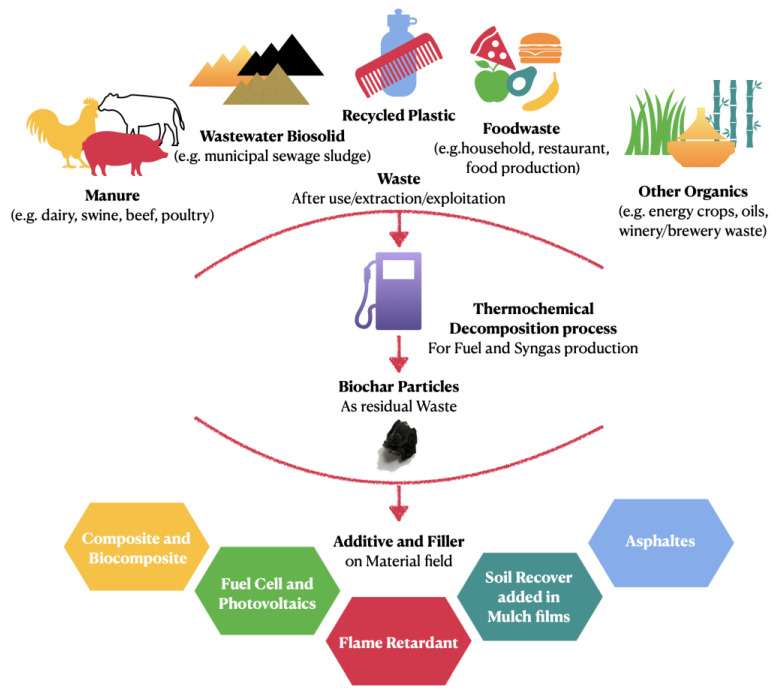
Different feedstocks used for the production of biochar particles and their adoption as suitable additives and fillers in polymers, biopolymers, and asphalts.

Therefore, this review reports on the use of biochar particles, coming from different sources, for the formulation of composites and asphalts. See Figure 1 for more detail. The first part of the review deals with the considered methods to produce biochar particles and their main properties; the second and thirst parts are related to the formulation of polymer-based and biopolymer-based composites, respectively; and the fourth part is focused on the use of biochar particles as new additives for asphalts systems.

## 2. Biochar Particles: Production, Characteristics, and Properties

An opportunity to convert solid/food waste and biomass includes the thermochemical decomposition processes that are being used with increasing frequency. The main thermochemical decomposition processes explored are slow, fast, and flash pyrolysis, torrefaction, gasification, and hydrothermal liquefaction. All of these processes essentially generate: *i.* a solid phase, named char or biochar (in the case of biomass feedstock), *ii.* fuel, a mixed liquid phase of the heaviest hydrocarbon, *iii.* syngas, and a mixed gas phase of the lightest hydrocarbons are produced [3,4,5,6]. Of course, depending on the chemical composition of the treated materials (i.e., biomass, mixed waste, synthetic polymers), and depending on the operative condition (i.e., temperature process, heating rate, presence or absence of oxygen, residence time), the relative ratio between these three main products could change. Slow pyrolysis, conducted in the absence of oxygen, is characterized by slow heating rates and long residence times, as well as atmospheric pressure with an operating temperature that can vary from 350 to 800 °C; the necessary energy to pyrolyze the feedstock is usually provided internally by combusting a portion of the feedstock. The main product is a high-carbon solid char, and the coproducts are watery, low molecular weight liquid and a low energy combustible gas [7,8,9,10]. Fast pyrolysis, like slow pyrolysis, is conducted in the absence of oxygen with a temperature range between 400 and 600 °C. In contrast to slow pyrolysis, it uses a very high heating rate under a vacuum atmosphere, a short residence time, and the rapid quenching of vapor, since the main goal of this process is to produce bio-oil [7,11,12]. Flash pyrolysis is a batch process with an operative temperature range between 300 and 800 °C, and is similar to slow pyrolysis but with a high heating rate that uses moderate pressure (between 2 and 25 atm) to condense volatile elements and to promote secondary formation, since the aim of this process is to produce a biocarbon liquid fraction or biochar solid phase [13]. Torrefaction is a slow pyrolysis method with a lower temperature range, between 200 and 300 °C, that mainly removes water and some volatiles from the biomass to produce a “brown” char that is easy to ground and is a stabilized and friable biomass. Gasification is characterized by a high process temperature (between 750 and 1800 °C) with a limited and controlled oxygen concentration (normally calculated as the amount relative to stoichiometric combustion) [14] and/or steam [15]; as the name suggests, the primary products are a non-condensable gas mixture, called syngas, which is essentially composed by the presence of CO, H_2_, with a smaller amount of carbon dioxide, methane, and other low molecular weight hydrocarbons [16]. Lastly, hydrothermal liquefaction is a process conducted in the presence of water, with a 250–450 °C temperature range under 100–300 bar; the main product of this process is called bio-crude, which is an energy-dense intermediate renewable source equivalent to oil that can be fractionated to a variety of liquid fuels [17]. Under this thermochemical process, the biomass is involved in depolymerization reactions (hydrolysis, dehydration, or decarboxylation), which produce insoluble products, such as bio-crude oil or bio-carbon, as well as volatile components (CO_2_, CO, H_2_ or CH_4_) or soluble organic substances (mainly acids or phenols). All these processes and their differences are summarized in Table 1.

The focus of this work is biochar, which is essentially a carbon-made material that can potentially be produced through any thermochemical process, as a primary or auxiliary co-product, and from any feedstock. Feedstocks could include building materials, agricultural waste, forestry residues, municipal solid waste etc. 

Biochar could be described as being divided into a “carbon” fraction, which includes carbon, hydrogen, and oxygen bonded together in different forms, and an ash inorganic fraction. For each thermochemical process employed, the temperature process, heating rate, and residence time affect the quality and the quantity of primary products and auxiliary co-products, and an operative parameter needs to be tailored to the feedstock, since the composition of potential biochar results may be affected by the feedstock characteristics. The primary analysis normally performed to characterize the feedstock is the operative temperature, and the relative char quality is the proximate analysis. This thermogravimetric analysis gives information about feedstock moisture content relative to the mass lost until 110 °C; volatile matter relative to the mass lost in an inert atmosphere at 950 °C; fixed carbon relative to mass lost in the air at 750 °C; and the remaining part relative to ash amount. Elemental analysis is normally employed to characterize the quality of char in terms of carbon content. This is a technique in which a sample is combusted at a very high temperature in a little chamber with an excess oxygen content, and the gasses relative to the combustion are trapped and, depending on the number of sensors available, it is possible to have, in terms of percentage in weight, information about carbon, hydrogen, nitrogen, CHN element amount (relative CO_2_, H_2_O, NO), sulfur content, CHNS, oxygen/sulfur contents, and CHNOS.

As discussed above, biochar is a carbon-rich material which can be prepared from various waste feedstock. Municipal solid waste and agricultural waste are only two examples of the many organic wastes that may be utilized as feedstock to create biochar. Sludge is a solid waste that must be treated and disposed of, since it is produced during the wastewater treatment process. However, because it includes abundant carbon and nutrients such as ammonia, it is a viable feedstock for the synthesis of biochar [18]. The high carbon content, high cation exchange capacity, vast surface area, and stable structure of biochar are only a few of its benefits [19].

In general, organic or synthetic material can be used as feedstock with different processes depending on the physiochemical characteristics and the product composition.

The value of a particular type of biomass depends on the chemical and physical properties of the molecules from which it is made. Biomass is the main feedstock used in the literature for BC production because of different advantageous reasons. First, for environmental reasons, biomass is more readily available in a renewable way, either through natural processes or as a product of human activities. Furthermore, when produced by sustainable means, biomass produces approximately the same amount of carbon during conversion as is taken up during plant growth, which reduces the CO_2_ amount in the atmosphere [20].

Biomass is mainly composed of three different organic compounds: cellulose, hemicellulose, and lignin, which give different mechanical and physiochemical properties to the woods. Cellulose makes up between 40% and 50% of the weight of dried wood and gives the biomass its strength [21]. Hetero polymers coexist with cellulose in plant cell walls to form hemicellulose. They contain several sugar monomers, including glucose, mannose, galactose, and xylose, and have lower molecular weights than glucose. Hemicellulose makes up anywhere from 20% to 35% of the bulk of dried wood [22]. The secondary cell wall of plants is made of lignin, which is a complex chemical compound. It is a kind of cross-linked resin that is amorphous, and it accounts for 15% to 30% of the mass of hardwoods. Depending on how much cellulose, hemicellulose, and lignin they contain, various biomass feedstocks have variable volatile matter concentrations and heating values, as well as different feedstock properties [23,24].

Biochar has received increasing attention due to its specific characteristics, such as high carbon content, cation exchange capacity, large specific surface area, and stable structure.

With different types of feedstocks, biochar has different physiochemical characteristics. The most typical processes for producing biochar are pyrolysis, gasification, and hydrothermal carbonization. Acid, alkali, oxidizing substances, metal ions, carbonaceous compounds, steam, and gas purging can all modify biochar. The environmental application fields determine the modification techniques to use.

The primary method used by biochar to remove organic and heavy metal contaminants is adsorption. The physiochemical characteristics of biochar, such as surface area, pore size distribution, functional groups, and cation exchange capacity, are strongly related to its adsorption ability, whereas physiochemical characteristics alter according to the production circumstances [25].

In general, biochar produced at high temperatures has a higher surface area and carbon content, mainly due to the increase in micro-pore volume caused by the removal of volatile organic compounds [26]. However, biochar yields decrease with temperature increases [27]. Therefore, an optimal strategy is required in terms of biochar yields and adsorption capacity. To sum up, the direct chemical composition of products and bioproducts is strictly connected to operative conditions (i.e., temperature, pressure and heating rate), which depend on the thermochemical process employed.

The physiochemical characteristics of biochar have been adjusted using metal ions, acids, alkalis, and oxidizing agents to make them better for various environmental processes [28].

Biochar has been widely employed in environmental applications, such as soil remediation, carbon sequestration, water treatment, and wastewater treatment because of its unique properties, which include high surface area, recalcitrant, and catalysis.

Common wastes, such as sludge and agricultural wastes, are produced in great quantities in the world. Sludge production alone reached 6.25 million tons in 2013 in China [29].

Converting common household wastes into biochar could be an option for environmental sustainability. Different feedstock has different proportions of element composition, and thus exhibits different properties, so the biochar derived from different feedstocks has various performances. The ways to deal with these wastes are directly linked to the impact they have on the environment.

Distinct feedstocks show varied qualities due to the different proportions of their elemental makeups, and, as a result, the biochar produced from those feedstocks performs differently. For instance, the pH (9.5) and potassium content (961 mg kg^−1^) of straw-derived biochar were greater than those of wood biochar (349 mg kg^−1^) [30]. Additionally, the biochar made from straw had more volatile material than non-volatile material, which is easier to remove during the pyrolysis process. Therefore, the high volatile component of the feedstock may contribute to poor biochar yields. Additionally, the content of pig and cow manures differed in terms of proportions [31]. Moreover, volatile content can be more easily removed than non-volatile content during pyrolysis. Therefore, the feedstock containing a high content of volatile content may result in low yields of biochar.

The type of feedstock has a significant effect on the physiochemical properties of biochar [32]. Therefore, the content of carbon in biochar is an important parameter, and different feedstocks can be converted into char using thermochemical decomposition processes, as was already described before (see Table 2). 

Due to different compositions (carbon with the presence of alkali metals, e.g., Li, Na, and K or alkaline metals, e.g., Ca, Mg, and Ba metals) depending on the nature of the feedstock, biochar can have versatile properties leading to many applications, including bioenergy (co-gasification, co-firing, and combustion), chemical use (as a catalyst or catalyst support), agronomy (regarding water retention, plant nutrients, or soil conditioner), pharmacological use (regarding the adsorption of drugs and toxins), environment remediation (regarding carbon sequestration and the sorption of pollutants), and as biomaterials for the production of bio-composites, fuel cells, and photovoltaic plants [46].

## 3. Polymer-Based Composites Containing BCp

As was already explained above, the final properties of BC particles depend on several factors, such as the nature of feedstock used to produce BC particles, the type of process employed, and the relative operative condition. The final content of fixed carbon and ash (which involves the milling and sieving process used to control the dimension of the final particles), their surface area, and pore volume consequently determine the final properties of the composites, and this also depends on the polymer matrix employed in terms of interfacial adhesion, dispersion, thermal and mechanical stability, and ageing protection efficiency. In order to assess how the pyrolysis temperature and type of feedstock could determine a difference in the final properties of composites, Das et al. [47] added biochar particles as a co-filler for producing wood plastic polypropylene-based composites. The authors also pyrolyzed different feedstocks (landfill pine sawdust, sewage sludge, and poultry litter) at different temperatures, with the aim of identifying a routing process for waste employing. In fact, when keeping the landfill pine wood weight percentage constant at 30 wt.%, 24 wt.% of biochar particles were obtained through the following procedures: *i.* pine wood was pyrolyzed through a two-step parallel reactor chamber with a retention time of 60 min at 900 °C for a high-temperature activation regime (TCP 900); *ii.* using the same reactor, a torrefaction regime reached pyrolyzing at 350 °C (TCP 350); *iii.* the same feedstock was pyrolyzed by means of an auger reactor with a retention time of 10 min at 470 °C (PSD470); *iv.* the same pilot plant was used for the same feedstock with the same retention time at 420 °C (PSD420); *v.* sewage sludge biochar was produced with a pyrolysis temperature of 680 °C and a retention time of 10 min; *vi.* biochar from chicken litter was produced at 450 °C and 20 min of residence time. The fixed biochar concentration of 24 wt.% was determined thanks to a previous work of the same research group [48], in which BC produced from pine wood was added to the PP matrix at different loadings that ranged from 6 to 30 wt.%. In that study, a BC content of 24 wt.% showed a general improvement in tensile and flexural strengths, as well as in the young modulus of the final composite. Taking into account these results, it was found that an increase in tensile strength and the moduli was strongly related to the increase in surface area. Moreover, the presence of residual minerals (i.e., CACO_3_ found in BC from chicken litter and the relative ash content) increased the impact strength of the composite and exhibited a lower heat release rate under the combustion regime compared to other composites. These results can be explained by considering that inorganic particles generally hinder the diffusion of oxygen through the matter, which creates a physical barrier between the combustible and the oxidizing agent, thus allowing the BC composites a possibility of being used in a flame-retardant field. Additionally, thanks to maleic anhydride grafted polypropylene/maleated anhydride polypropylene (MAPP) being used as a coupling agent, a general good dispersion of BC particles and an infiltration of polypropylene into biochar pores was observed for all composites. Without wood presence for the PP-based composites, a flame-retardant ability in pine wood biochar was established through a study by Das et al. [49], in which various BC loads, from 0 to 35 wt.% of the composites, exhibited increasingly stable compact char structures during controlled combustion tests that hid the O_2_ diffusion in a polypropylene matrix. Moreover, the addition of char significantly reduced peak heat release and smoke production. The increase in flame retardant ability conferred by the presence of biochar particles in wood polypropylene composites was also explored in the presence of conventional inorganic flame retardants, such as magnesium hydroxide and ammonium polyphosphates [50,51]. On the other hand, in the presence of biochar, the two flame retardants particles were trapped into BC pores instead of in polypropylene with a final reduction in PP flow during processing and consequent reduction in interfacial adhesion and relative mechanical stability. Furthermore, the addition of biochar particles in wood polypropylene composites bestowed the composite with resistance toward water. This result remained valid without exceeding a threshold concentration, up to which the composites became more susceptible to water. In addition, it has been found that high pyrolysis temperatures generate more hydrophilic particles, due to absorption through the capillary action of pores [52]. Moreover, the reason for adding biochar particles to a Wood Polypropylene Composite (WPC) is that WPC usually suffers of thermal instability and thickness swelling, due to the high hydrophilic behavior of wood dust. Ayrilmis et al. [53] progressively reduced wood dust concentrations from 60 to 0 wt.% while respectively increasing commercial *Quercus* char flour concentrations from 0 to 60 wt.%, wherein thickness swelling was reduced by the 50% after 30 days when wood dust was completely substituted by BC dust, which increased the global dimensional stability. The same stabilization behavior was established for the water absorption after 30 days of WPC substation with BC dust, which decreased from ca. 21% for the PP/wood composite to ca. 15% for the PP/char composites. The same result of dimensional stabilization and global improvement of resistance to thermal degradation by adding BC particles to WPC was also confirmed through a study conducted by DeVallance et al. [54,55]. A variation in final composite properties related to pyrolysis temperature was also highlighted in a polypropylene/poly (octene-ethylene) copolymer (POE) (70/30 wt.%) blend [56], in which 10 and 20 wt.% of high-temperature pyrolyzed biocarbon (HTBioC) and low temperature pyrolyzed biocarbon (LTBioC) from *Miscanthus* were added. The HTBioC showed a lower presence of functional groups on the char surface, as well as a higher porosity with a relative increase in surface area that promoted better compatibility to the polymer blend, while also having a significantly better stiffness–toughness balance in the composite compared to the LTBioC.

Giorcelli et al. [57] also studied the relationship between the pyrolysis temperature and the electrical conductivity of biochar particles with the intention to use biochar particles as a filler in epoxy resin-based conductive composites. The residues of *Miscanthus* were pyrolyzed at 650, 700, and 750 °C and activated by CO_2_ to increase surface area. The residues were characterized, and, then, 20 wt.% of the particles were added into epoxy resins for electrical characterization. As was already shown by other studies, it was found that an increase in pyrolysis temperature corresponded to and increase in carbon content with a corresponding reduction in other elements (i.e., O, Mg, Si, K, Ca) [46], and the ratio between the disordered and graphitized structure of the carbon structure increased with the increase in pyrolysis temperature. All of these properties led to an increase in the conductivity of biochar particles as a function of pyrolysis temperature, with a consequent increase in electrical performance for composites obtained with the addition of particles produced at higher pyrolysis temperatures. In the same lox viscosity epoxy resin LPL (Cores Ocean), two different biochars obtained by pyrolyzing Maple tree waste at low and high temperatures (600 and 1000 °C) were added at different weight percentages in order to improve the mechanical properties of the resin [58]. It was observed that the addition of a small amount of carbon fillers, lower than 2 wt.%, increased the load bearing capacity of the epoxy matrix, while also modifying the mechanical properties of the polymer matrix; on the other hand, a concentration equal to or higher to 2 wt.% transformed the pristine epoxy resin from brittle to a ductile composite, which was different from what was already seen for polypropylene-based composites. The optimum filler level depends on the type of polymer, the pyrolysis temperature, the type of feedstock used for biochar production, and the presence of other additives in composite production. When adding a curing agent (i.e., cycloaliphatic polyamine) and an embedding medium during epoxy-based composite processing, it is possible to increase the filler content above a critical level that normally lowers tensile strength. This configuration was found at a critical level of chars obtained from natural substances equal to 25 wt.%, and, in composites with plastic waste char, the critical level appeared to be reasonably low, equal to 15 wt.% [59]. Nevertheless, plastic waste char, or PWC (made from the pyrolysis of polyethylene terephthalate, PET), due to the terephthalic acid in the char structure, increased the global conductivity of a polymer composite [60].

Another way to activate biochar particles has been explored by Zhang et al. [61] by means of the impregnation of biomass feedstock before the carbonization process in an H_3_PO_4_ solution. In that work, biochar from rice husk, obtained pyrolyzing at 600 °C, was compared with activated biochar by varying the H_3_PO_4_ concentration in the activation solution. Generally, the activation of BC improved the thermal stability of the resulting composites, but a different concentration of activating agent affected the characteristic of the biochar in terms of chemical and morphological structure. In fact, a low concentration of H_3_PO_4_ improved the porous structure, which improved the resulting mechanical properties, thanks to better adhesion between particles and the HDPE polymer matrix, including flexural properties, rigidity elasticity, creep resistance, and anti-stress relaxation. On the other hand, a high concentration of H_3_PO_4_ in the activation solution generated fouling in the porous structure, which reduced all mechanical properties.

To further improve electrical properties, a carbonization process of charcoal from three different biomasses [62] has been performed with high fill ultra-high molecular weight polyethylene/linear low density poly ethylene UHMWPE/LLDPE [63]. For example, starting with charcoal coming from bamboo pyrolysis, which was further carbonized at 1100 °C in a muffle furnace in the absence of air, particles with irregular shapes have been obtained with a global transformation of their amorphous structure into a graphite-like structure with a higher crystallinity grade. This result was simultaneously confirmed by an increase in the three diffraction peaks at around 24.6°, 43.7°, and 50.1°, which were associated with the C (002), C (100), and C (004) diffractions, respectively, of the graphitic structure through XRD analysis and with an increase in the intensity ratio of the D-to-G peak obtained through Raman spectroscopy, which revealed a defective graphitic structure and turbostratic crystallites in the BC1100 particles. Moreover, the high temperature carbonization generated a high specific surface, due to the creation of a nanoporous structure. These particles were added into a UHMWPE blended with a LLDPE as a flow accelerator for reducing the melt viscosity of the UHMDPE and improving the processability of the composite and final particle dispersions. The highly filled composites (with a carbon load up to 80 wt.%) showed excellent electromagnetic interference shielding performance, and one of the highest values reported for conductive polymer composites was found; in fact, at maximum biochar concentration, a conductivity of 107.6 S/m was found. The same further-carbonized bamboo charcoal particles were used with high fill UHMWPE to produce scaffolds for cells proliferation. In that study [64], the raw bamboo charcoal and bamboo charcoal carbonized at 800 °C and 1100 °C were over pyrolyzed in a muffle furnace in the absence of air. Additionally, in that case, thanks to high temperature and the relative development of the surface into a nanoporous structure, crystallinity, hardness, and thermal stability were found to be higher for the biochar carbonized at the higher temperature. On the other hand, for better biocompatibility, achieving a high temperature of pyrolysis is not useful, because, at low temperature, biochar enables the composite to exhibit better hydrophilicity and higher specific surface energy, which promotes protein adhesion and cell proliferation. Moreover, globally, the composite obtained by adding to the UHMWPE showed good mechanical properties and friction performance that make it appropriate for use in orthopedic applications.

Arrigo et al. [65] performed an extra carbonization process of a torrefied coffee powder was into a tubular furnace, and the waste was pyrolyzed at 700 °C for 1 h under a nitrogen atmosphere, which was then added to high density polyethylene, HDPE, to understand the interaction between HDPE and BC from spent coffee grounds, as well as how the filler content influenced the rheological and thermal behavior of the resulting composites. The authors subjected BC/HDPE composites with different BC loads (up to 7.5 wt.%) to SEM analysis and rheological characterization that employed different flow fields, including linear and non-linear dynamic shear flow, which resulted in clear confinement of the polymer chains onto the surface of particles and into the porous structure of particles, as well as a pseudo solid-like behavior of the BC/HDPE composites due to the formation of a network.

To reduce waste for environmental purposes, Kane et al. [66] recently compared recycled high-density polyethylene, rHDPE, with and without biochar to look at both improving mechanical properties and environmental impact. From a mechanical point of view, the tensile behavior of the rHDPE was significantly altered by the addition of biochar particles coming from wood forestry residues, which increased the strength and stiffness through the global increase of crystallinity of the rHDPE through the reinforcing action of the polymer matrix, and, thanks to a good interface adhesion, that led to a polymer interlocking with the porous structure of the biochar. Moreover, by means of life cycle assessment, it has been noticed that the addition of biochar as a filler reduces the global amount of plastic spent to produce a product, which of course provides a benefit in terms of global warming potential when referring to the CO_2_ emitted for plastic production. It has been calculated that rHDPE reached a 0 kg CO2 equivalent by adding less than 40 wt.% of biochar particles, which obtained a composite with a similar strength and stiffness obtained by adding 40–50 wt.% of biochar particles to virgin HDPE [67].

Another way to reduce the amount of polyolefin waste and reduce the amount of virgin polyolefin employed in the industrial field has been addressed in a study conducted by Idress et al. [68], in which a recycled poly-ethylene-terephthalate rPET-based composite was produced by adding biochar. The biochar employed in that work came from the high temperature pyrolysis (1100 °C) of PET waste under an autogenic pressure of ca. 150 bar. In that study, the researchers were able to extrudate recycled PET and PET/BC composites, which highlighted that the incorporation of biochar enhanced the mechanical properties and provided the PET with thermal properties, which suggests that BC could supply the necessity for commercial graphene materials in polymer composites. As was noticed with other polyolefins, the responsibility for the improvement in mechanical properties must be referred to the high surface porosity of BC particles and the high affinity between BC particles and the polymer matrix. The improvement in thermal properties must be referred to the known barrier effect of BC.

## 4. Bio-Polymer-Based Composites Containing BCp

**Figure 2:**
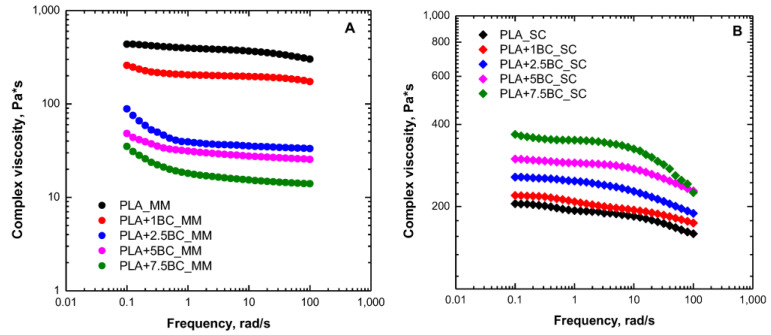
Complex viscosity as a function of frequency for neat poly(lactic acid) (PLA) and biochar (BC)-containing composites obtained through melt mixing (MM) (**A**) and solvent casting (SC) (**B**) [74].

Bioplastics should be intended as polymers that meet any of two criteria: the polymer is bio-based and/or biodegradable [69]. In the context of a sustainable and circular economy, the recovery of bio-waste and the addition of them in biopolymers, intended as bio-based and biodegradable, for sustainable bio-composites formulation is a challenging issue. Among biopolymer-based composites containing biochar particles, the literature reports a significant number of studies. One of the most biochar-added polyester matrixes is polylactic acid (PLA), which suffers from poor thermal stability and high brittleness that reduces its employment in many fields, i.e., textile, biomedicine, and food packaging [70]. Briefly, the use of BC in PLA can lead to growth in the PLA market, thanks to a global improvement in the mechanical stability of this polymer [33,71,72]. Kane et al. [73] investigated BC-added PLA composites and compared them to high density polyethylene HDPE composites. In contrast to HDPE composites, for PLA/BC composites, the work highlighted an impact of BC in thermal degradation behavior, which was shown through a decrease in onset degradation temperature and a global reduction in melt viscosity of the PLA, which was probably due to the presence of an inorganic element of the BC surface being responsible for catalyzing PLA thermal decomposition. The same behavior has been found by Arrigo et al. [74], in which BC particles derived from spent ground coffee were added in the PLA matrix by processing the composites through melt mixing and solvent casting methods. It was found that the PLA rheological behavior underwent significant alteration when the composites were obtained by melt processing. In fact, the authors reported (see Figure 2) a progressive increase in melt viscosity in composites obtained by solvent casting and a progressive decrease in melt viscosity in composites obtained by melt mixing, as the BC content increased, which, in the last case, suggested a severe reduction in polymer molar mass, due to thermal degradation [75], and PLA preservation when the processing was carried out at room temperature by means of solvent casting. In any case, a strong polymer-filler or filler-filler interaction has been found, which was demonstrated by the appearance of a yield stress behavior.

A significant reduction in the molecular mass of poly(3-hydroxybutyrate) (PHB) processed at high temperatures with the addition of biochar particles has been demonstrated by Haeldermans et al. [76]. In their study, different PHB/char with varying BC loads (from 20 wt.% to 50 wt.%) were produced, and their formulation was compared with PHB/thermoplastic starch (TPS)/BC composites. Despite having the best biodegradability compared to other biopolymers, it is well known in the literature that PHB suffers from a significant reduction in molecular weight after processing, and this limits its application, due to a really small operational processing window [77], e.g., an unprocessed PHB-M_w_ of 611 Kg/mol and a melt-processed PHB-M_w_ of ca. 463 Kg/mol [76]. Regrettably, increasing the amount of biochar particles from 20 to 50 wt.% further reduced the PHB-M_w_, which achieved a reduction to 218 Kg/mol for 50 wt.% of BC and reduced the global thermal stability of the BC bio-composites. Consistent with molecular weight reduction, a reduction in thermal properties was found, such as a decrease in melting point with a decrease in molecular weight [78]. Thanks to the presence of thermoplastic starch in bio-composites, the decrease in M_w_ is more gradual and controlled, and molecular weight analysis has shown that, at low BC loads, TPS can act as an intermediator between PHB and PHB by controlling the reduction in molecular weight.

In contrast, the addition of BC particles as a filler in an Ecovio commercial polymer blend containing poly(1,4-butylene adiphat-co-1,4-butylene terephthalate), PBAT, 47 mol% of an aromatic segment, and PLA, 25 mol%, significant increased the application field of BC particles in the biopolymers matrix [79]. In fact, a significant reduction in the resistivity of obtained bio-composites was found as the BC load increased by up to 30 wt.%, which suggests employment of the composites in equipment elements in laboratories for precise measurement, or as an antistatic agent in the packaging industry. Moreover, thermal stability has not been affected by the presence of BC compared to the Ecovio polymer matrix. Moreover, thanks to a global improvement of modulus shown in DMA analysis for all temperature ranges (−50 to 120 °C), mechanical properties were higher for composites with respect to the neat matrix, which suggested better mechanical stability.

**Figure 3:**
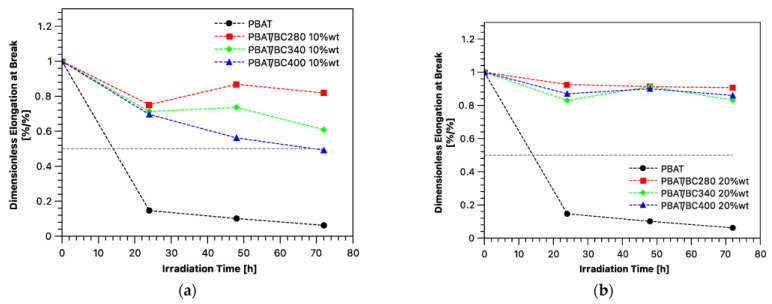
The trend of dimension elongation at break with (**a**) 10 wt.% and (**b**) 20 wt.% of filler content in the PBAT matrix [37].

Regarding the PBAT matrix containing biochar particles, several works have been published. Botta et al. [80] investigated the properties and the filmability of PBAT-based materials that were added to commercial biochar powder used in the food industry that was formed from birch and beech wood pyrolysis. The team performed a preliminary investigation of the prepared PBAT/BC composites by melt mixing with BC loads from 5 wt.% to 20 wt.% of commercial BC, which showed a uniform filler dispersion and a good adhesion within the selected biopolymer matrix, which led to an increase in global mechanical properties. Moreover, DSC analysis clarified how the BC did not influence the PBAT chain structure, which remained almost amorphous despite filler addition, even with the increase in T_m_ as the filler content increased and revealed compatibility between the filler and matrix. Instead, the rheological behavior of PBAT-based composite results were affected by the presence and the increase in carbonaceous filler, which resulted in a relative increase in melt viscosity, in all ranges of frequency, and suggested an influence of embedded filler on the long-range and short-range dynamics of polymer chains, especially when the BC load was equal to 20 wt.%. At that carbon load, the PBAT underwent a dramatic reduction in its intrinsic ductility and a significant decrease in the break–stretching ratio (BSR), which resulted in a composite with no filmability properties. The same rheological and mechanical behavior has been found by Infurna et al. [37], in which agricultural carob waste was pyrolyzed at three different temperatures (BC280, BC340, and BC400 respectively pyrolyzed at 280, 340, and 400 °C) and then added to PBAT at two different concentrations, i.e., 10 and 20 wt.%. In their work, an ageing protection assessment was performed on both the pristine particles and on the BC composites. First, the authors characterized the radical scavenging efficiency by means of 1,1-diphenyl-2-pycryl (DPPH) free radical analysis, in which the three different BC particles were added at constant loads to a methanol solution of DPPH, a stable free radical, and they monitored the disappearance of the free radical UV absorption peak at 517 nm. From their analysis, thanks to residual functional groups on the BC surface after 24 h, the particles obtained at a lower pyrolysis temperature achieved about 100% radical scavenging efficiency, despite the values obtained at 400 °C, with higher scavenging kinetics of the BC280. The monitoring of the DPPH UV absorption peak was also performed by increasing the amount of BC in the solution. In that case, it was demonstrated that, from a limited concentration onwards after 24 h, the radical scavenging efficiency results were comparable between the three different particles. This result is consistent with what has been found in the photooxidation assay of biopolymer composites, as partly shown in Figure 3, in which the variation in mechanical properties as a function of irradiation time was monitored while also extrapolating the half time as the time at which the elongation at break was half of the initial one.

A significant reduction in the ductility of pristine PBAT is shown, which achieved a half time of 14 h. In Figure 3a, the same trend of the DPPH assay is shown, in which the lower the pyrolysis temperature was of the obtained BC particles, the higher the concentration of functional groups on the surface were able to scavenge free radicals from the accelerating weathering test, which resulted in a higher shown resistance of the bio-composites. In conclusion, it was enough to increase the BC load from 10 wt.% to 20 wt.% to lead to a comparable ageing resistance for all bio-composites, and the same results have been found by ATR-FTIR analysis as a function of irradiation time.

Polyvinyl alcohol (PVA)/corn starch/BC bio-composites were successfully formulated by means of the solvent casing method in the presence of citric acid and glutaraldehyde, which was added for fixative effect before the casting period [81]. The interaction between the biopolymer blend and BC particles significantly affected the degradation path of the PVA/starch composites. In fact, a significant decrease in the narrowing of the peak relative to the hydroxy band with the increments of BC load was noticeable. The authors attributed this phenomenon to the good compatibility of the PVA, starch, and BC [82]. As had happened for PLA and PHB-based composites, the global thermal stability of the bio-composites was lower when BC was added to the blend.

A noticeable improvement in mechanical properties has been obtained by introducing a suitable concentration of biochar particles in eco-friendly bio-composites manufactured by a green epoxy matrix reinforced with short agave fibers for replacing synthetic materials in structural applications [83]. In that case, with the optimum amount of BC particles (in this case found to be equal to 2 wt.%) added by means of the synergic effect of short fiber and biochar particles, a better adhesion has been achieved between the epoxy matrix and fiber, which was demonstrated by the fiber pull-out test. This aspect involved an increase in the global Young’s modulus and tensile strength, as well as an increase in fatigue performance with an increase in fatigue strength by about 67% and fatigue lifetime by at least three orders of magnitude. This remarkable enhancement of mechanical performance increases the possibility of employing a green epoxy matrix fiber reinforced for structural and semi-structural applications, especially in automotive and naval applications.

Green composites have been formulated by adding biochar particles in partially bio-based polymers or bio-based polymers that are non-biodegradable. Nagarajan et al. [6] performed a study of the varying particle size distributions of biochar particles produced from the low temperature slow pyrolysis process of *Miscanthus* fibers. After pyrolysis, size-fractionation of the BC was performed with sieves having different openings, e.g., 300, 212, 150, 125, 75, and 20 μm. The different size-fractioned BCs were added to a poly(trimethylene terephthalate) (PTT) 70/poly(lactic acid) (PLA) 30- ethylene-methyl acrylate-glycidyl methacrylate terpolymer (EMAGMA) polymer blend (85–15), using, in some formulation, an epoxy-functionalized chain extender (CE). A good range of particle size distribution, combined with the presence of the chain extender, helped to obtain a morphology with a better dispersion of the blend components, i.e., particle size distributions of 75–20 μm. Biochar particles under 20 μm in size diameter stabilized the blend morphologies, which showed a coalescence of PLA-EMAGMA particles that turned in smaller and finer morphologies in the presence of CE. This evaluation obtained from SEM image observations was consistent with rheological tests and mechanical analysis, which showed that, with an appropriate BC particle size and shape, morphologies and properties can be tailored to achieve desired properties with solid cost reduction. The partially bio-based PTT was combined with 20 wt.% high temperature bio-carbon from peanut hull pyrolysis, which resulted in superior mechanical performance that could be optimized for non-structural automotive components or electrical housing applications [84]. Also in this case, particle size distribution and particle size of the original biomass can play a crucial role in the resulting biochar, impact the concentration of volatiles and bio-oil from the pyrolysis process [85,86] and, as expected in bio-composite properties, call for a milling process before pyrolysis has been conducted [84]. The addition of peanut hull biochar, with its sheet-like surface morphology with high graphitic carbon content and its relatively low electrical conductivity, can contribute to the improved thermal stability of PTT-based green composites by increasing both flexural and tensile moduli, which suggest nonstructural and anti-static applications.

## 5. Asphalts Composites Containing BCP

Using biochar particles as an asphalt binder modifying filler is going to become a new and interesting application field. In fact, nowadays in the construction sector, it is already used as a substitute for cement in mortar or concrete, thanks to its help with accelerating cement hydrating [87] and due to global CO_2_ mitigation [88]. The new approach regards the addition of asphalt binders as an ageing protector, which are an essential component of asphalt concrete in addition to being the heaviest coproduct of the petroleum refining system, after distillation, to obtain fuels and lubricants [89]. The oxidation of asphalt binders is an inevitable phenomenon that plays an enormous role in the deterioration of asphalt binders. In fact, the life expectancy of usual binders is susceptible to ultraviolet (UV) rays, which cause faster oxidation of asphalts, with a sensitive reduction in rheological characteristics and a loss of rutting properties, which can lead to pavement distress [90]. Walters et al. [91] investigated the impact of added biochar particles (coming from a thermochemical process used to convert swine manure in bio-oil) or nano-clay (Cloesite 30B) on the rheological properties and ageing susceptibility of asphalt binder, and compared the results with a control asphalt (PG 64–22) binder. The introduction of BC to the asphalt binder led to a reduction in asphalt temperature susceptibility, and, regarding the shear susceptibility, its sensitivity decreased by adding 10 wt.% of BC to PG 64–22, which achieved a lower value than control asphalt. Contrary to the addition of BC, the addition of nano-clay generated an impact on the layer spacing, which appeared to be responsible for enchaining the high temperature performance and ageing resistance of asphalt binders. In a second study by Walters et al. [90] a composite with both biochar (3 wt.%) particles and nano-clay (3 wt.%) was produced that resulted in a lower viscosity than the ones with only nano-clay, while the ageing susceptibility was improved significantly. This happens because biochar seems to have a role in the flow modifiers alleviating the stiffening effect of nano-clay, which help the nano-clay to disperse better in an asphalt binder.

Zhao et al. [92] evaluated the properties and performance of asphalt binders and mixtures by adding 5 wt.% and 10 wt.% of biochar from the fast pyrolysis of switchgrass for biofuel production. In their work, the authors found that biochar significantly increased the rutting resistances at high service temperatures of both asphalt binders and asphalt mixtures. Moreover, the addition of 5 wt.% of biochar may be the optimum in modifying binders in terms of cracking resistance, while 10 wt.% shows little effect in comparison with 5 wt.%. Another study was conducted by the same research group by adding different BC particles to a commonly used asphalt binder (PG 64–22), which resulted in a different type of pyrolysis of switchgrass, while taking into account that BC results as a by-product for biofuel production, and comparing these lab-made BC particles with a commercially activated carbon [93]. In particular, the BC particles were produced by the following techniques: *i.* a microwave reactor in which switchgrass was mixed with silicon carbide to absorb enough microwaves, then the mixture was heated up until 500 °C in less than 1 min and the temperature was maintained for 15 min, and, then, after cooling down, the silicon carbide particles were sieved out to finally obtain BC particles with size diameters between 75 and 150 μm; *ii.* a tube furnace method in which feedstock was heated up until 400 or 500 °C with a heating rate of 15 °C/min to result in BC particles with diameters smaller than 75 μm for particles obtained at 400 °C and 500 °C, and for the ones obtained at 500 °C a diameter range between 75 and 150 μm was obtained as well; *iii.* an activated commercially available carbon was selected for comparison. The composites were characterized in terms of viscosity modification, ageing and fatigue resistance, and rutting properties of un-aged and aged composites. The addition of all bio-modifiers increased the viscosity of the asphalt binder at a high service temperature and exploited a positive effect in ageing resistance at a long time of UV exposition. Globally, except for particles with smaller diameters (<75 μm) produced at 400 °C that showed a positive effect on the specific properties or performance of the asphalt binders, the pyrolysis method appeared to have a negligible effect on the degree of modification. Another study published by Zhang et al. [94] focused on varying biochar loads and biochar diameter distributions by comparing the biochar contribution on varying properties with the ones obtained by graphite adding. The biochar used in the work was obtained from waste wood resources, at a temperature ranging from 500 and 650 °C, through a pyrolysis plant able to heat at 10 °C/s. Then, BC particles were sieved to separate the different size ranges, and the size range of previous work was studied, i.e., between 75 and 150 μm and lower than 75 μm; the biochar contents in PG 58–28 control asphalt were 2 wt.%, 4 wt.%, and 8 wt.%, respectively. The flake graphite with a diameter lower than 75 μm and content of 4 wt.% was also added to PG 58–28 for comparison. As was expected, BC addition resulted in higher porosity and micro-structure compared with dense and smooth graphite. This aspect of course led to a larger and better adhesion interaction in the asphalt binders of BC than that of graphite, and, as a result, BC modified binders had better high-temperature rutting resistance and better anti-ageing properties, especially for the BC-modified binder with a lower BC diameter at a higher content.

In conclusion, it seems that the addition of BC to asphalt seems to increase the thermal resistance during asphalt preparation and their oxygen resistance in service. This is another important result of asphalt reducing viscosity during processing, which helps the dispersion of other asphalts constituents in order to obtain a high-performance pavement.

## 6. Conclusions

Char/biochar particles could be considered as a new kind of sustainable particle created from their “waste” feedstocks. Specifically, when also considering the circular principles, the conversion of polymer waste, food waste, and biomasses, through thermal treatment at high temperatures, gives an appropriate second life for these waste materials. Produced biochar particles differ in their physical and chemical properties, e.g., CHNOS compositions, porosity, and adsorption ability, because of the implementation of different thermal treatments, such as slow, fast, and flash pyrolysis; torrefaction, gasification; and hydrothermal liquefaction. Currently proposed applications of the new char/biochar particles are mainly as follows: *i.* as fertilizers for soil retirement, *ii-* as high adsorption particles for water remediation, and *iii.* as suitable fillers for the formulation of polymer/biopolymer-based composites and to produce asphalts. Therefore, this review critically reports on the current status of char/biochar particles in considering the main advantages and drawbacks in the use of these new particles as suitable fillers for polymers, biopolymers, and asphalts.

The char/biochar particles, being particles mainly composed of carbon atoms and having a large surface, are very useful to formulate composites with improved mechanical resistance, i.e., elastic modulus and tensile strength, as well as improved oxidative and photooxidative resistance, while also considering the particles’ radicals scavenging abilities in comparison to the properties of neat matrices. Unfortunately, the main drawback is related to the particle color. Particularly, being black particles, their composites appear mainly with brawn-black coloration, and, obviously, this is a limitation for large scale esthetic applications.

## Figures and Tables

**Table 1:** Thermochemical processes and their main differences in terms of operative conditions, time of reactions, and primary products.

Thermochemical Process	Temperature Range [°C]	Heating Rate	Pressure	Residence Time	Primary Product
Slow Pyrolysis	350–800	Slow (<10 °C/min)	Atmospheric	Hours—Days	Char
Fast Pyrolysis	400–600	Very Fast (~1000 °C/s)	Vacuum-Atmospheric	Seconds	Oil
Flash Pyrolysis	300–800	Fast	Moderate (2–25 atm)	Minutes	Biocarbon/ Char
Torrefaction	200–300	Slow (<10 °C/min)	Atmospheric	Minutes—Hours	Friable Biomass
Gasification	700–1800	Moderate-Vary Fast	AtmosphericModerate	Seconds—Minutes	Syngas/ Producer gas
Hydrothermal Liquefaction	250–450	Moderate	Elevated 100–300 atm	Minutes—Hours	Bio-crude (oil)

**Table 2:** Proximate analysis of different biomass raw materials and relative elemental composition after pyrolysis process.

Feedstock	Proximate Analysis			Elemental Analysis			
	Volatile Matter [wt.%]	Fixed Carbon [wt.%]	Ash [wt.%]	C [wt.%]	H [wt.%]	N [wt.%]	O [wt.%]
Alfalfa [33] (*Medicago sativa)*	78.90	15.80	5.30	49.90	6.30	2.80	40.80
Almond Shell [34]	74.90	21.80	3.30	50.30	6.20	1.00	42.50
Bagasse [35]	71.00	13.70	2.10	51.71	5.32	0.33	42.64
Bamboo [36]	81.60	17.50	0.90	52.00	5.10	0.40	42.50
Carob Waste [8,37]	38.80	52.80	3.80	46.94	1.63	5.44	-
Coconut Fiber [38]	80.85	11.10	8.05	47.75	5.61	0.90	45.51
Corncob [39]	69.50	15.90	2.90	48.12	6.48	-	43.51
Cornstalk [39]	65.30	15.60	11.70	46.21	6.01	-	45.87
Cocopeat [35]	49.10	25.30	4.60	61.57	4.37	1.02	33.04
Dead Eucalyptus leaves [40]	77.60	16.90	0.80	52.90	8.10	0.30	47.90
Hamlin citrus [33]	77.90	17.80	9.40	50.70	6.60	1.60	42.90
Hornbeam Shell [41]	78.83	9.37	9.52	41.78	5.36	0.60	52.26
Loblolly Pine [33] (*Pinus taeda)*	77.60	14.50	2.30	55.50	5.60	0.40	45.90
Maize straw [42]	-	-	5.31	42.20	7.21	1.28	49.20
Mesocarp Fiber [40] (*Oil palm*)	72.80	18.90	8.30	51.50	6.60	1.50	40.10
Oak sawdust [43]	69.24	16.51	0.81	52.28	5.74	0.06	41.92
Olive wood [44]	79.60	17.20	3.20	49.00	5.40	0.70	44.90
Paddy straw [35]	56.40	15.40	20.90	48.75	5.98	1.99	43.28
Pinewood [38]	85.45	13.15	1.40	48.15	6.70	1.35	43.60
Palm Kernel Shell [35]	66.80	17.90	3.40	55.82	5.62	0.84	37.73
Raw Pine sawdust [42]	83.10	16.80	3.76	50.60	6.18	0.05	43.10
Rice husk	62.80	19.20	18.00	49.30	6.10	0.80	43.70
Sawdust [39]	70.40	18.50	1.20	48.37	4.98	-	46.27
Tea waste [45]	70.29	18.57	3.88	48.60	5.43	3.80	42.17
Wood Stem [35]	80.10	10.70	0.40	50.52	5.81	0.23	43.44
Wood Bark [35]	68.90	16.30	4.90	53.42	6.12	1.40	39.06

## Data Availability

Not applicable.
